# Effects of first- and second-generation tyrosine kinase inhibitor therapy on glucose and lipid metabolism in chronic myeloid leukemia patients: a real clinical problem?

**DOI:** 10.18632/oncotarget.5580

**Published:** 2015-09-10

**Authors:** Alessandra Iurlo, Emanuela Orsi, Daniele Cattaneo, Veronica Resi, Cristina Bucelli, Nicola Orofino, Mariarita Sciumè, Chiara Elena, Valeria Grancini, Dario Consonni, Ester Maria Orlandi, Agostino Cortelezzi

**Affiliations:** ^1^ Oncohematology Division, IRCCS Ca’ Granda – Maggiore Policlinico Hospital Foundation, and University of Milan, Milan, Italy; ^2^ Oncohematology Unit of the Elderly, IRCCS Ca’ Granda – Maggiore Policlinico Hospital Foundation, and University of Milan, Milan, Italy; ^3^ Endocrinology and Diabetes Unit, Department of Medical Sciences, IRCCS Ca’ Granda – Maggiore Policlinico Hospital Foundation, and University of Milan, Milan, Italy; ^4^ Oncology-Hematology Department, Hematology Unit, Fondazione IRCCS Policlinico San Matteo, Pavia, Italy; ^5^ Epidemiology Unit, Department of Preventive Medicine, IRCCS Ca’ Granda – Maggiore Policlinico Hospital Foundation, Milan, Italy

**Keywords:** chronic myeloid leukemia, imatinib, dasatinib, nilotinib, diabetes mellitus, metabolic syndrome

## Abstract

**Background:**

Tyrosine kinase inhibitors (TKIs) have dramatically changed the prognosis of patients with chronic myeloid leukemia (CML). They have a distinct toxicity profile that includes glycometabolic alterations: i.e. diabetes mellitus (DM), impaired fasting glucose (IFG), and the metabolic syndrome (MS). The aim of this study was to evaluate the prevalence of these alterations in a cohort of CML-chronic phase patients treated with imatinib, dasatinib or nilotinib.

**Methods:**

The study involved 168 consecutive CML-chronic phase patients with no history of DM/IFG or MS. Anthropometric and metabolic parameters were assessed, and DM/IFG and MS were diagnosed based on the criteria of the American Diabetes Association and the National Cholesterol Education Program-Adult Treatment Panel III, respectively.

**Results:**

The nilotinib group had significantly higher levels of fasting plasma glucose, insulin, C-peptide, insulin resistance, and total and LDL cholesterol than the imatinib and dasatinib groups. DM/IFG were identified in 25% of the imatinib- and dasatinib-treated patients, and 33% of those in the nilotinib cohort (*p* = 0.39 vs imatinib and *p* = 0.69 vs dasatinib). A diagnosis of MS was made in 42.4% of the imatinib-treated patients, 37.5% of the dasatinib-treated patients, and 36.1% of the nilotinib-treated patients (*p* = 0.46 vs imatinib and *p* = 0.34 vs dasatinib).

**Conclusions:**

Treatment with nilotinib does not seem to induce DM/IFG or the MS to a significantly higher extent than imatinib or dasatinib, though it causes a worse glycometabolic profile. These findings suggest the need for a close monitoring of glucose and lipid metabolism and a multidisciplinary approach in patients treated with nilotinib.

## INTRODUCTION

Until 1999, drug therapy for chronic myeloid leukemia (CML) [1, 2] was limited to non-specific agents such as busulfan, hydroxyurea, cytosine arabinoside, and interferon-α (IFN-α) [3]. The development of small-molecule tyrosine kinase inhibitors (TKIs) has dramatically changed patients’ prognosis as they potently interfere with the interaction between the *BCR-ABL1* protein and adenosine triphosphate (ATP), and block the proliferation of the malignant clone [4]. This “targeted” approach has significantly changed the natural history of CML and improved 10-year overall survival from less than 20% to 80-90% [2, 5]. Imatinib mesylate was the first TKI to be approved by the US Food and Drug Administration for the treatment of patients with CML-chronic phase, followed in 2007 by the second-generation TKIs dasatinib and nilotinib [6–11].

TKIs approved for first- and second-line treatment of CML-chronic phase have a distinct toxicity profile that includes glycometabolic alterations such as diabetes mellitus (DM), impaired fasting glucose (IFG), and the metabolic syndrome (MS), a cluster of metabolic abnormalities characterized by insulin resistance [12]. Based on its age distribution, it can be predicted that prevalence of CML will increase with the increasing age of the general population, and that this will lead to a significantly higher risk of developing these metabolic disorders upon treatment with certain TKIs. In fact, a large phase III trial comparing the efficacy of nilotinib and imatinib showed that hyperglycemia occurred in 50% of patients treated with nilotinib 300 mg b.i.d., 53% of those treated with nilotinib 400 mg b.i.d., and only 31% of those treated with imatinib 400 mg/day; yet, none of these patients discontinued TKI therapy because of hyperglycemia or had serious diabetes-related adverse events [13]. However, no data are available concerning the prevalence of DM/IFG and the MS in “real-life”, unselected CML patients on TKI therapy.

Purposes of this study were (a) to assess the prevalence of glycometabolic alterations (DM/IFG, MS) in a cohort of CML-chronic phase patients on TKI therapy; and (b) to identify which parameter(s) should be evaluated at diagnosis and during treatment to help clinicians to choose the most appropriate TKI for each patient from a metabolic point of view.

## RESULTS

One hundred and sixty-eight consecutive patients diagnosed as having CML-chronic phase and treated with imatinib (*n* = 92), dasatinib (*n* = 40) or nilotinib (*n* = 36) entered the study. Among them, 107 were in first-line, 53 in second-line, and the remaining 8 in third-line treatment. Moreover, only 3 patients changed TKI because of intolerance to the previous treatment, whereas all the other patients were resistant. Our cohort included 92 males (54.8%), and their median age at the time of recruitment was 56.0 years (range 21.2-87.5) (Table 1).

**Table 1:** Clinical and laboratory features of 168 CML-chronic phase patients treated with imatinib, dasatinib or nilotinib

	Imatinib group (n. 92)	Dasatinib group (n. 40)	Nilotinib group (n. 36)	P-value*
Age (years), median (range)	59.2 (21.2-87.5)	54.9 (24.5-82.6)	46.0 (25.1-79.0)	0.0044
Male/Female, n	49/43	23/17	20/16	0.90
Length of treatment (months), median (range)	93.0 (0.4-165.5)	41.6 (3.0-97.1)	16.1 (0.1-70.0)	0.0001
BMI (Kg/m^2^), mean (SD)	25.9 (4.8)	24.8 (3.7)	26.3 (3.5)	0.24
FPG (mg/dl), mean (SD)	98.0 (22.8)	92.8 (12.0)	104.7 (18.9)	0.0069
HbA_1c_ (%), mean (SD)	5.5 (0.7)	5.5 (0.9)	5.6 (0.6)	0.54
Insulin (μUI/ml), median (range)	7.9 (2.3-33.1)	6.7 (2.7-29.8)	11.0 (6.2-29.5)	0.0001
C-peptide (μmol/L), median (range)	2.3 (1.1-8.5)	1.9 (0.9-4.2)	2.8 (1.8-4.8)	0.0003
HOMA-IR, median (range)	1.7 (0.6-12.0)	1.4 (0.6-7.9)	2.8 (1.4-7.0)	0.0001
HOMA-%B, median (range)	94.3 (22.8-550.8)	92.3 (42.1-364.1)	99.7 (34.9-393.2)	0.26
Total cholesterol (mg/dl), median (range)	177.0 (119.0-265.0)	189.0 (114.0-337.0)	217.5 (140.0-297.0)	0.0001
HDL cholesterol (mg/dl), median (range)	48.0 (20.0-98.0)	50.0 (23.0-109.0)	53.0 (32.0-87.0)	0.09
Triglycerides (mg/dl), median (range)	102.0 (32.0-506.0)	111.0 (46.0-457.0)	104.0 (36.0-249.0)	0.59
LDL cholesterol (mg/dl), mean (SD)	105.2 (32.1)	124.2 (35.4)	140.0 (36.5)	0.0001
SBP (mmHg), median (range)	130.0 (100.0-180.0)	122.5 (100.0-170.0)	125.0 (110.0-150.0)	0.28
DBP (mmHg), mean (SD)	79.0 (7.8)	77.9 (8.8)	80.1 (6.5)	0.39
Waist circumference (cm), mean (SD)	94.3 (15.7)	90.4 (12.8)	94.2 (12.2)	0.40
DM/IFG, n (%)	23 (25.0)	10 (25.0)	12 (33.3)	0.61
MS, n (%)	39 (42.4)	15 (37.5)	13 (36.1)	0.77

CML= chronic myeloid leukemia; BMI= body mass index; FPG= fasting plasma glucose; SD= standard deviation; HbA1c= hemoglobin A1c; HOMA-IR= Homeostasis Model Assessment – Insulin Resistance; HOMA-%B= Homeostasis Model Assessment – β-cell function; SBP=systolic blood pressure; DBP= diastolic blood pressure; DM= diabetes mellitus; IFG= impaired fasting glucose; MS= metabolic syndrome.

* From Kruskal-Wallis (continuous variables) or chi-squared (categorical variables) test.

In detail, at the time of assessment, patients in the imatinib group were significantly older and were receiving treatment for longer than those in the other groups. There were significant differences in fasting plasma glucose (FPG), insulin, C-peptide, and the Homeostasis Model Assessment - Insulin Resistance (HOMA-IR) index, but not in body mass index (BMI), waist circumference, hemoglobin (Hb) A_1c_ and HOMA - β-cell function (HOMA-%B). Moreover, there were significant differences in total and LDL cholesterol, but not in HDL cholesterol, triglycerides, and systolic and diastolic blood pressure.

Table 2, 3, and 4 show the results of the multiple regression analyses adjusted for centre, gender, age, BMI and length of treatment, and expressed as absolute differences or percent changes. FPG, insulin, C-peptide, and HOMA-IR were all significantly higher in the nilotinib group than in the other two groups, whereas there was no difference in HOMA-%B. Total, HDL, and LDL cholesterol levels did not differ significantly between the dasatinib and nilotinib groups, but they were significantly higher in both groups than in the imatinib group, except for HDL cholesterol between dasatinib- and imatinib-treated individuals. No difference in triglyceride levels was found among the three groups.

**Table 2:** Multiple regression analysis adjusted for centre, gender, age, BMI, and length of treatment, comparing imatinib versus nilotinib group

	Imatinib group (n. 92)	Nilotinib group (n. 36)	
		Coefficient (95% CI)*	P-value*
FPG (mg/dl)	Reference	9.8 (1.9; 17.8)	0.015
Insulin (%)	Reference	17.4 (6.2; 30.3)	0.002
C-peptide (%)	Reference	11.6 (4.1; 19.7)	0.002
HOMA-IR (%)	Reference	22.1 (9.4; 37.7)	<0.001
HOMA-%B (%)	Reference	2.0 (−9.5; 10.5)	0.76
Total cholesterol (%)	Reference	11.6 (7.3; 16.2)	<0.001
HDL cholesterol (%)	Reference	6.2 (1.0; 11.6)	0.02
Triglycerides (%)	Reference	3.6 (−5.8; 13.9)	0.48
LDL cholesterol (mg/dl)	Reference	42.4 (27.0; 57.8)	<0.001
		OR (95% CI)**	P-value**
DM/IFG	Reference	1.7 (0.5; 5.8)	0.39
MS	Reference	0.6 (0.2; 2.4)	0.46

BMI= body mass index; CI= confidence interval; OR= odds ratio; FPG= fasting plasma glucose; HOMA-IR= Homeostasis Model Assessment – Insulin Resistance; HOMA-%B= Homeostasis Model Assessment – β-cell function; DM= diabetes mellitus; IFG= impaired fasting glucose; MS= metabolic syndrome.

* From multiple linear regression models.

** From multiple logistic regression models.

**Table 3:** Multiple regression analysis adjusted for centre, gender, age, BMI, and length of treatment, comparing dasatinib versus nilotinib group

	Dasatinib group (n. 40)	Nilotinib group (n. 36)	
		Coefficient (95% CI)*	P-value*
FPG (mg/dl)	Reference	9.7 (2.5; 16.9)	0.009
Insulin (%)	Reference	22.1 (11.6; 33.6)	<0.001
C-peptide (%)	Reference	16.2 (9.4; 24.0)	<0.001
HOMA-IR (%)	Reference	27.1 (16.2; 40.5)	<0.001
HOMA-%B (%)	Reference	7.3 (−4.9; 20.9)	0.27
Total cholesterol (%)	Reference	3.9 (−0.8; 8.3)	0.10
HDL cholesterol (%)	Reference	3.0 (−2.0; 8.3)	0.24
Triglycerides (%)	Reference	−5.8 (−14.8; 4.1)	0.23
LDL cholesterol (mg/dl)	Reference	14.8 (−2.5; 32.1)	0.09
		OR (95% CI)**	P-value**
DM/IFG	Reference	1.3 (0.4; 4.1)	0.69
MS	Reference	0.5 (0.2; 1.9)	0.34

BMI= body mass index; CI= confidence interval; OR= odds ratio; FPG= fasting plasma glucose; HOMA-IR= Homeostasis Model Assessment – Insulin Resistance; HOMA-%B= Homeostasis Model Assessment – β-cell function; DM= diabetes mellitus; IFG= impaired fasting glucose; MS= metabolic syndrome.

* From multiple linear regression models.

** From multiple logistic regression models.

**Table 4:** Multiple regression analysis adjusted for centre, gender, age, BMI, and length of treatment, comparing imatinib versus dasatinib group

	Imatinib group (n. 92)	Dasatinib group (n. 40)	
		Coefficient (95% CI)*	P-value*
FPG (mg/dl)	Reference	0.2 (−4.9; 5.2)	0.95
Insulin (%)	Reference	−3.9 (−12.2; 5.7)	0.42
C-peptide (%)	Reference	−4.4 (−10.4; 2.0)	0.18
HOMA-IR (%)	Reference	−3.9 (−13.1; 7.3)	0.48
HOMA-%B (%)	Reference	−4.9 (−13.9; 6.2)	0.37
Total cholesterol (%)	Reference	7.8 (4.1; 11.6)	<0.001
HDL cholesterol (%)	Reference	3.0 (−2.0; 8.3)	0.22
Triglycerides (%)	Reference	9.4 (0.2; 20.9)	0.046
LDL cholesterol (mg/dl)	Reference	27.6 (14.7; 40.5)	<0.001
		OR (95% CI)**	P-value**
DM/IFG	Reference	1.3 (0.4; 4.2)	0.60
MS	Reference	1.1 (0.4; 3.5)	0.85

BMI= body mass index; CI= confidence interval; OR= odds ratio; FPG= fasting plasma glucose; HOMA-IR= Homeostasis Model Assessment – Insulin Resistance; HOMA-%B= Homeostasis Model Assessment – β-cell function; DM= diabetes mellitus; IFG= impaired fasting glucose; MS= metabolic syndrome.

* From multiple linear regression models.

** From multiple logistic regression models.

On the basis of clinical and laboratory data, 45 patients (26.8%) were classified as having type 2 DM/IFG and 67 (41.4%) as having the MS, with no significant between-treatment group differences at both univariate (Table 1) and multiple regression analyses (Table 2, 3 and 4). Only 2 patients (1.2%) discontinued TKI therapy because of the development of glycometabolic alterations, whereas 3 subjects (1.8%) required a specific treatment for hyperglycemia, without discontinuing TKI therapy.

## DISCUSSION

Each of the TKIs approved for treatment of patients with CML-chronic phase has a distinct toxicity profile that should be carefully considered when prescribing TKI treatment. It is now well known that first- (imatinib) and second- (dasatinib, nilotinib) generation TKIs act on different molecular targets [14], and that their side effects are mainly due to the inhibition not only of *BCR-ABL1* but also of other tyrosine kinases such as *c-kit*, *PDGFR*, *Src* or *EPHB4*. Imatinib mainly causes peripheral edema, whereas dasatinib predisposes some patients to pleural effusions and, to a lesser extent, pulmonary arterial hypertension [15], and inhibits platelet function [16, 17]. Both drugs do not negatively impact on glucose metabolism and may even improve FPG levels and allow a reduction of the dosage of anti-hyperglycemic drugs [18–21]. The positive effect of imatinib might be due to an improvement of both insulin secretion, via inhibition of β-cell apoptosis [22] due to increased activation of antiapoptotic transcription factor *NF-kB* or decreased activation of the proapoptotic *MAPK JNK*, and insulin sensitivity in peripheral tissues, via inhibition of tumour necrosis factor-α production [23] and/or endoplasmic reticulum stress [24, 25]. Conversely, nilotinib was shown to induce hyperglycemia in a subset of non-diabetic CML patients [13, 26, 27]. The underlying mechanism is controversial as a previous analysis of 10 patients demonstrated a reduction in insulin sensitivity [28], whereas a case report showed a reversible decrease in insulin secretion [29] under nilotinib therapy. Regarding lipid profile, total, HDL, and LDL cholesterol levels were found to increase significantly in patients treated with nilotinib, and to decrease in those receiving imatinib [30]. The latter effect might be due to the fact that imatinib can act also on *PDGFR*, which may induce phosphorylation of LDL receptor-related protein [31].

Our cross-sectional analysis assessed the prevalence of DM/IFG and the MS in a large series of “real-life”, unselected CML-chronic phase patients during TKI treatment and estimated both insulin sensitivity and β-cell function in these individuals. We found a non-significant trend toward an increase in the prevalence of DM/IFG, but not the MS, in CML-chronic phase patients treated with nilotinib *vs* those receiving imatinib or dasatinib. Moreover, individuals on nilotinib showed significantly higher FPG, insulin, C-peptide, and total and LDL cholesterol levels and HOMA-IR values, with no differences in HbA_1c_ and HOMA-%B.

The increase in FPG, but not in HbA_1c_ levels, in patients treated with nilotinib suggests that the hyperglycemic effect of this drug may not be clinically meaningful and that it likely does not affect post-prandial glucose concentrations. This interpretation is consistent with the higher HOMA-IR, but not HOMA-%B values, indicating an impairment of insulin sensitivity with no effect on β-cell function. Also the lack of significant increases in the prevalence of DM/IFG observed in patients receiving nilotinib, which is at variance with a previous report from a phase III trial [13], seems to argue against the concept that the effect of this drug on glucose metabolism represents a real clinical problem. This view is consistent with a previous report that fasting hyperglycemia does not progress to type 2 DM in nilotinib-treated individuals [27]. However, our prevalence data might have been influenced by a selection bias caused by the significant inter-groups differences in age and duration of TKI treatment, which may have not been accounted for by statistical adjustment. In fact, patients on nilotinib were younger and with a shorter treatment duration than those on imatinib and dasatinib. Taken together, our data, while excluding a potent diabetogenic effect of nilotinib, do support the need for a close monitoring of glucose (and lipid) metabolism in patients receiving this drug and for a multidisciplinary approach to manage rises of FPG (and cholesterol) levels during treatment. In clinical practice, it would be appropriate to measure FPG, cholesterol, and possibly HbA_1c_ levels before treatment to identify subjects at risk to develop glycometabolic abnormalities and to guide the choice of the TKI. Moreover, the same parameters should be assessed at 3 months of treatment, and in the nilotinib group every 6-12 months thereafter. This strategy would be adequate for reducing cardiovascular burden associated with hyperglycemia and dyslipidemia, which may increase the risk of atherosclerosis with peripheral artery occlusion occurring with nilotinib treatment [30, 32, 33].

Strengths of our study include the “real-life” setting and the size of the cohort, which is much larger than in previous reports [18–21, 26]. Limitations include the cross-sectional design of the study, the already mentioned differences among treatment groups in terms of age and duration of TKI therapy, and the criteria for excluding the presence of DM and the MS at baseline. In addition, the fact that an oral glucose tolerance test was not performed in the study subjects might have resulted in an underestimation of the prevalence of DM.

In conclusion, this cross-sectional analysis of CML-chronic phase patients on treatment with three TKIs showed that prevalence of DM/IFG or the MS was not significantly different, though glycometabolic profile was worse in subjects treated with nilotinib than in those on imatinib or dasatinib. These findings indicate the need for a close monitoring of glucose and lipid metabolism and a multidisciplinary approach in patients treated with nilotinib.

## MATERIALS AND METHODS

### Patients

One hundred and sixty-eight consecutive CML-chronic phase patients attending the Oncohematology Division of the IRCCS Ca’ Granda – Maggiore Policlinico Hospital Foundation, Milan, and the Hematology Unit of the IRCCS Policlinico San Matteo Hospital Foundation, Pavia, were recruited between May 2013 and July 2014. The study was approved by the local Ethics Committees, and all patients gave their written informed consent in accordance with the Italian guidelines for the protection of human subjects. Inclusion criteria were age >18 years, CML-chronic phase diagnosis based on World Health Organization 2008 criteria, and current treatment with imatinib, dasatinib or nilotinib [14]. CML patients who received only IFN-α therapy were excluded from the analysis, as were those with a previous history of DM/IFG, MS, or statin treatment. FPG levels and body weights before starting TKI treatment were also available in all cases.

### Methods

Anthropometric and metabolic parameters of each patient were assessed at enrollment. All patients underwent physical examination, with assessment of BMI, waist circumference, and blood pressure, and biochemical testing, including HbA_1c_, FPG, insulin, and C-peptide levels, and serum lipid profile.

As a surrogate measure of insulin secretion, the HOMA-%B was calculated from fasting insulin and glucose concentrations by the formula: (20xIns_0_)/(Gluc_0_-3.5). The HOMA-IR index was estimated using the formulas proposed by Levy *et al*. [34, 35].

Diabetes mellitus, IFG, and the MS were diagnosed according to the criteria of the American Diabetes Association, updated in 2010 (based on FPG and HbA_1c_ levels) [36], and those of the National Cholesterol Education Program - Adult Treatment Panel III [12].

### Statistical analysis

The Kruskal-Wallis and chi-squared tests were used to compare continuous and categorical variables, respectively, in the treatment groups. The laboratory results were evaluated by fitting multiple linear regression models adjusted for centre, gender, age, BMI, and length of treatment. Two sets of comparisons were made: 1) nilotinib and dasatinib *vs* imatinib; and 2) nilotinib *vs* dasatinib. In the case of FPG and LDL cholesterol, the regression coefficients represent absolute differences (in mg/dL). Conversely, as the other laboratory data were right skewed, we fitted regression models using their log_10_-transformed values as dependent variables, and then calculated their percent changes using the formula: % change = [antilog_10_(Coefficient) – 1]×100 [37]. For DM/IFG and the MS, we used logistic regression models to calculate adjusted odds ratios (ORs) and 95% confidence intervals (CIs), once again using the imatinib or dasatinib groups as reference [38]. The statistical analyses were made using Stata 13 software [39].
